# Metabolic effects of dietary exposure to polystyrene microplastic and nanoplastic in fruit flies

**DOI:** 10.1242/jeb.250522

**Published:** 2025-10-13

**Authors:** Eric A. Riddell, Rachel M. Sorensen, Elizabeth McNeill, Boris Jovanović

**Affiliations:** ^1^ Department of Ecology, Evolution, and Organismal Biology, Iowa State University, Ames, IA 50011, USA; ^2^ Department of Biology, University of North Carolina, Chapel Hill, NC 27599, USA; ^3^ Department of Food Science and Human Nutrition, Iowa State University, Ames, IA 50011, USA

**Keywords:** *Drosophila*, Plastic particle toxicity, Polystyrene, Metabolic rate, Water loss rate, Water loss, Metabolism

## Abstract

Understanding how anthropogenic change impacts metabolic physiology is crucial for predicting species survival and ecosystem dynamics. Microplastics are ubiquitous in both aquatic and terrestrial environments and can disrupt organismal physiology. We used *Drosophila melanogaster* as a model species to identify the metabolic effects of dietary exposure to 1 µm polystyrene microplastic (MP) and 50 nm nanoplastic (NP) particles. We exposed flies to ecologically relevant and equivalent doses (1.4×10^11^ particles day^−1^ kg^−1^ larvae for MPs; 1.2×10^18^ particles day^−1^ kg^−1^ larvae for NPs) from egg to adult eclosion and used flow-through respirometry to investigate changes in the volume of carbon dioxide production and evaporative water loss rate. We observed that MP exposure disrupted the relationship between carbon dioxide production and water loss rate – suggesting the use of alternative metabolic pathways – while NP exposure did not. Such responses could have implications for physiological function, ecological interactions and evolutionary trajectories amid ongoing environmental change.

## Teaser

**Summary:** Polystyrene microplastics, but not nanoplastics, disrupt fruit fly energetics by weakening CO_2_–water loss coupling and inflating metabolic variability.

## INTRODUCTION

In a rapidly changing world, understanding how environmental stressors affect the metabolic physiology and overall performance of organisms is important for predicting species survival and potential cascading effects throughout ecosystems (Norin and Metcalfe, 2019). Organisms rely on finely tuned metabolic processes to maintain energy balance, grow and reproduce as they cope with diverse challenges such as climate change, habitat disruption and chemical exposures (Seebacher et al., 2010). One emerging concern is the pervasive presence of small plastic particles in both aquatic and terrestrial environments. These particles, which range in size from microplastics (MPs; <5 mm) to nanoplastics (NPs; 1–100 nm), have been found to infiltrate food webs and even accumulate in human tissues (Roslan et al., 2024). Research on aquatic organisms has shown that these plastics can disrupt physiological functions by triggering inflammation, oxidative stress and endocrine disruption (Arthur et al., 2009; Greven et al., 2016; Jovanović, 2017). Thus, a better understanding of small plastic particle effects is essential for predicting impacts on natural systems.

Our understanding of the impact of MPs and NPs on terrestrial organisms remains limited, especially for insects. Numerous studies have assessed the toxicity of MPs and NPs in various terrestrial species (Buteler et al., 2022; de Souza et al., 2022; Huerta Lwanga et al., 2016; Liu et al., 2023; Sherlock et al., 2022; Hohman et al., 2024), yet for insects, studies have been mostly restricted to aquatic taxa (Al-Jaibachi et al., 2018; Gopinath et al., 2022; Nel et al., 2018; Stanković et al., 2020; Yildiz et al., 2022). Given the critical roles that insects play in pollination, decomposition and the integrity of food webs, there is an urgent need to explore how dietary exposure to these particles influences metabolic function. Such studies might be particularly insightful when conducted on well-studied model organisms (such as *Drosophila melanogaster*), which might also help us to understand the effects of plastic ingestion in human food sources (Diaz-Basantes et al., 2020; Karami et al., 2017; Kosuth et al., 2018; Neves et al., 2015). In this study, we investigated the effects of polystyrene because, with 2.1 million metric tons produced in 2018 and only 0.9% recycled (EPA, 2020a,b), its extensive use in food packaging leads to significant environmental accumulation. By examining these effects, our research not only advances our understanding of plastic particle toxicity but also provides insights into how environmental contaminants might broadly influence metabolic physiology and performance across species (Sorensen et al., 2023).

In our study, we focused on the metabolic rate of *D. melanogaster*, a well-established model organism for studying metabolic function (Herranz and Choen, 2017; Lim and Mathuru, 2018; Pandey and Nichols, 2011). In *Drosophila*, previous studies have noted the presence of MPs in the gut and body (Alaraby et al., 2022a,b; Matthews et al., 2021; Kholy and Al Nagger, 2023; Tu et al., 2023), reductions in cardiovascular function (Chen et al., 2020; Hohman et al., 2024), reductions in food intake (Shen et al., 2021; Kholy and Al Naggar, 2023; Zhong et al., 2022) and shorter life spans (Tang et al., 2023). However, the potential impacts of MPs and NPs on metabolic rate or pathways in energy metabolism remain largely unexplored. In other organisms, plastic exposure can disrupt glucose metabolism (Qiao et al., 2022), potentially suggesting organisms might have reduced metabolism or use alternative metabolic pathways for energy (such as lipids). In this study, we used flow-through respirometry to determine whether dietary exposure to polystyrene MPs and NPs reduced metabolic rates of flies or disrupted the close association between metabolic rate and water loss rate (Lehmann, 2001). Metabolic rates and water loss rates are closely associated across terrestrial life because the conditions necessary for gas exchange (both oxygen uptake and carbon dioxide elimination) are also responsible for high rates of water loss, thereby producing a fundamental trade-off between the capacity to breathe and the capacity to remain hydrated (Woods and Smith, 2010; Riddell et al., 2024). The association has also been observed in *Drosophila* (Lehman et al., 2000; Lehman, 2001) and potentially played essential roles in adaptation to abiotic conditions (Addo-Bediako et al., 2001; Gibbs et al., 2003). We hypothesized that dietary exposure to polystyrene MPs and NPs would lower metabolic rate and potentially disrupt the well-established relationship between metabolic rate and water loss rate (Lehmann, 2001; Gibbs et al., 2003; Woods and Smith, 2010). Such responses could have implications for physiological function, ecological interactions and evolutionary trajectories in the face of anthropogenic environmental change.

## MATERIALS AND METHODS

### Test species, materials and diet

We obtained *D. melanogaster* type W^1118^ from stock cultures (stock no. 83009, Bloomington Stock Center, Bloomington, IN, USA). We selected W^1118^ flies because of their shorter lives under starvation and oxidative stress compared with controls (Ferreiro et al., 2018), two possible endpoints of MPs and NPs exposure. Also, W^1118^ flies have previously been used in NPs/MPs research (Tu et al., 2023; Hohman et al., 2024; Sorensen et al., 2024). Further, we chose a standard laboratory strain to reduce genetic variability that might obscure physiological responses. We maintained the W^1118^ culture at 25°C in 60% humidity with a 12 h light/dark cycle. We raised the colony of flies on a standard diet consisting of cornmeal, sugar, yeast (Lewis, 1960; Ward's Science, Rochester, NY, USA, catalog number 470303-066, CAS# 68876-77-7), agar with Tegosept (0.2% of final solution, Genesse Scientific, El Cajon, CA, USA, catalog number 20-258, CAS# 99-76-3) and 2-propanol (0.4% of final solution) as mold inhibitors. We purchased polystyrene NPs and MPs from Phosphorex Inc. (Hopkinton, MA, USA, catalog numbers 102 and 112, respectively, CAS# 9003-53-6). MPs were 1.051±0.199 μm in diameter, while NPs were 0.041±0.007 μm in diameter (sample mean±s.d.), as specified by the manufacturer. Particles were spherical in shape and were suspended in ultra-pure deionized (DI) water with 0.1% Tween 20 to prevent clumping.

**Fig. 1.**
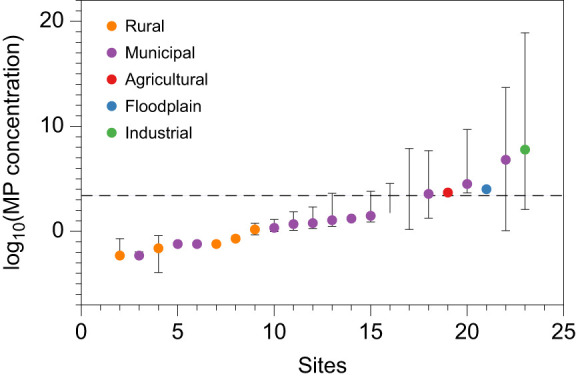
**Ecological relevance of plastic particle exposure.** The figure illustrates the log_10_-scaled concentration of microplastics (MPs) in soil samples (organized by concentration) as reported in Buks and Kaupenjohann (2020) and Yang et al. (2021). The horizontal dashed line corresponds to the concentration used in our study, assuming the dry mass of fly media. Concentrations are reported in mg of plastics per kg of dry soil. Colors correspond to the site classification (e.g. rural, municipal, etc.). Points represent the average concentrations, and bars indicate the range of values observed at each site. Missing lower bars indicate lower range values were zero.

Following standard protocols (Lewis, 1960), we prepared the diet by first melting the agar in water, and then adding cornmeal, dextrose, yeast and sucrose mixed in water. After reaching the correct consistency, the mixture was removed from the heat and allowed to cool to less than 65°C in order to ensure that the plastic would not melt. Then, we pipetted 2.5 ml of either vortexed MPs/NPs stock solution or water (control diet) into the solution and immediately stirred. Instant dry yeast was sprinkled on every bottle before the addition of flies. The final concentration of Tween 20 was 0.000002%, which was also added to the control diet group. The final number of particles was 1.4×10^11^ particles day^−1^ kg^−1^ of larvae for MPs and 1.2×10^18^ particles day^−1^ kg^−1^ of larvae for NPs. Following the methods of Jovanović et al. (2018), *Drosophila* larvae were exposed to 0.0783 g plastic kg^−1^ larvae day^−1^ for both MPs and NPs – a dose 7.8 times greater than the estimated maximum daily consumption for a 70 kg human (0.01 g kg^−1^ body mass) (Senathirajah et al., 2021). This dose (0.0783 g plastic kg^−1^ larvae day^−1^ or approximately 0.0008% of their body mass in plastics per day) was selected as a worst-case scenario in terms of human concentration exposure (Senathirajah et al., 2021). This dose corresponds to a concentration of 0.0047% (47 ppm) in the total fly media, or ∼0.0254% when calculated based on the dry components alone. In the environment, plastic particle concentrations are highly variable; therefore, we compared the concentration of MPs in our study with those reported in two recent reviews (Büks and Kaupenjohann, 2020; Yang et al., 2021). We observed that our treatments reflect ecologically relevant concentrations in soil that animals might experience, particularly in more industrial or urbanized landscapes (Fig. 1).

Before transferring flies to treatments, all flies were anesthetized with CO_2_ and sorted using FlyPad and a binocular scope. We added 15 male and 15 female flies (from the stock culture) to three 177.4 ml bottles of each diet (MP, NP and control). We allowed flies to mate for 2 days, and on the third day, we placed females in new bottles according to their respective diets. We allowed females to lay eggs for 8 h before removing and discarding them. Larvae that hatched from these eggs developed on their respective diets through adult eclosion. Thus, the experimental flies were exposed to their treatment throughout both larval development and a portion of their adult life.

### Metabolic rate

We measured the volume of carbon dioxide production (*V̇*_CO_2__) and evaporative water loss (EWL) rates using a flow-through system capable of precise control over flow rates, temperature and humidity. The flow-through system consisted of a pump [PP4, Sable Systems International (SSI)] that pumped air from a carboy into a dewpoint generator (DG4, SSI) to control the vapor pressure entering the animal chamber. We maintained vapor pressure at 0.95 kPa (roughly 30% relative humidity) to minimize exposure to desiccation (Gibbs et al., 2003). The air then passed into a manifold and flow bar (FB8, SSI), which split the airstream and regulated the flow rate at 25 ml min^−1^, and then into the glass chamber (∼10.1 ml in volume) containing the flies. Small amounts of cotton at the ends of the chamber were used to inhibit the flies from escaping through the tubing. The glass animal chambers were located inside a temperature-controlled incubator (ReptilePro 6000) and maintained at a constant temperature of 25°C. A multiplexing device (MUX, SSI) was used to cycle between different chambers. From the multiplexer, the air then passed into a vapor pressure analyzer (RH-300, SSI) and a carbon dioxide analyzer (CA10, SSI). Each of these analyzers was then used to calculate EWL rates and *V̇*_CO_2__. Each instrument was zeroed using research-grade nitrogen immediately prior to the experiment, and the instruments were zeroed and spanned using CO_2_ and H_2_O-free air, and the dewpoint generator and research grade CO_2_, respectively. These steps help to minimize potential sources of error related to drift and incorrect calibrations.

After preliminary testing, we determined that 20 flies were sufficient to reliably measure the signature of carbon dioxide production. Specifically, the signal was approximately 10-fold greater than the noise or better, thereby minimizing error. To quantify the physiological differences between fly treatments, we measured the metabolic rate and EWL rate of 20 flies in each chamber that had been exposed to NPs, MPs or control media (no plastic). Flies were measured after 12 days post-initial egg exposure. Each respirometry trial consisted of a chamber representing each treatment, and we randomized the order in which treatments were measured using a random number generator. We measured metabolic rate and EWL rate at night to increase the chance that flies were resting and mostly inactive in a dark environment. Prior to the measurements, flies rested in the metabolic chamber for 1 h, and then each chamber was measured 3 times over a period of ∼6.5 h. This measurement period is similar to that in previous studies conducted on *Drosophila* (Gibbs et al., 2003). Before the experiment, we measured the mass of the 20 flies by measuring the mass of the chamber with and without flies to the nearest 0.001 g. The possible differences between sex were minimized by controlling the number of males and females in each chamber (9–11 males and females in each chamber). We used parallel experimental setups to ensure that all flies were analyzed when they were 12 days old (since deposited as eggs). There were a total of 5 groups of 20 flies for each treatment (100 flies per treatment), which we measured 3 times for a total of 45 measurements for *V̇*_CO_2__ and EWL across the entire experiment (15 measurements per treatment group) (see Fig. S1 for a diagram of the experiment).

For data analysis, we used standard protocols and calculations to determine *V̇*_CO_2__ and EWL (Lighton, 2008). We used ExpeData (v.1.9.27, SSI) to visualize and calculate metabolic rate and EWL rate. All measurements were stable and exhibited low variance, indicating flies were resting. The pipeline included lag correction, drift correction using a Catmull–Rom spline, and water vapor compensation. We used standard procedures and calculations for EWL and *V̇*_CO_2__ using eqns 3 and 4 in Riddell et al. (2017) and eqn 11.8 in Lighton (2008), respectively. We recorded the mean values for each physiological rate across each measurement period for the respective treatments. EWL was expressed as mg h^−1^, whereas the units for *V̇*_CO_2__ were ml min^−1^.

We note that the *V̇*_CO_2__ measurements in this study are highly comparable to those reported in previous studies (Gibbs et al., 2003; Videlier et al., 2021) once corrected for the number of individuals and units (average *V̇*_CO_2__=0.000864 ml min^−1^ per 20 individuals; average mass-specific *V̇*_CO_2__=0.0448 ml min^−1^ g^−1^). Similarly, the EWL measurements are consistent with those of other studies (average EWL=0.229 mg h^−1^ per 20 individuals; average mass-specific EWL=11.89 mg h^−1^ g^−1^), after accounting for differences in ambient humidity (Gibbs et al., 2003; Lehmann, 2001), which is typically lower than that used in our experiments.

### Statistical analysis

We analyzed metabolic rate (*V̇*_CO_2__) and water loss rate (EWL) using linear mixed-effects models in R (v.4.0.2) using the *lme4* package. We ensured all models met the assumptions for normality and homoscedasticity using a Shapiro–Wilk test and by assessing residuals, respectively. We also noted limited evidence for collinearity using variance inflation factors (VIF<10). We first conducted analyses on *V̇*_CO_2__ and EWL separately. These models included the physiological trait as the response variable, treatment (control, NP and MP) as a factor, and mass (g) of the 20 flies as a covariate to properly account for the effects of mass (Hayes, 2001). Each model also accounted for repeated measures on groups of flies using the chamber ID as a random effect. We also assessed the well-established relationship between metabolic rate and water loss rate in a separate analysis, with metabolic rate as the dependent variable and mass, treatment and water loss rate as predictors. The analysis also included the interaction between EWL and treatment to determine whether the relationship between metabolic rate and water loss rate differed among treatment groups. We used Type-II ANCOVA with Satterthwaite's degrees of freedom from the *lmerTest* package to determine significance. We also conducted pairwise *post hoc* comparisons (i.e. Tukey) using the *emmeans* package. We also conducted a *post hoc* Levene's test to determine whether variances in metabolic rate differed between treatment groups after accounting for effects of mass and water loss. Because of the relatively low sample size, we also report effect sizes using omega-squared (ω^2^), which describes the total variation explained by predictors (Olejnik and Algina, 2003), helping to assess the practical importance of the effect beyond statistical significance. We also used power simulations using the powerCurve() function in the *simr* package (Green and MacLeod, 2016) to estimate the number of replicates (that is, chambers of flies) with 95% confidence intervals (95% CI) that would be required to detect the observed effects of our predictors with 80% power. This analysis was intended to clarify whether non-significant *P*-values were primarily due to limited statistical power or to a genuinely small treatment effect (that is, Type-II errors). To illustrate the effects of predictors, we plotted the adjusted means derived from the statistical model, representing the response variable after controlling for other covariates. All figures were made using DataGraph™ (v.5.4).

## RESULTS AND DISCUSSION

Exposure to small plastic particles did not significantly affect metabolic rate or water loss rate when analyzed in isolation (*P*≥0.551 for all comparisons). For both traits, we observed no detectable effect of treatment (ω^2^=0.00) (Tables S1 and S2). Using the power analysis, we observed that we would need extremely high sample sizes to detect an effect of treatment (metabolic rate: 127, 95% CI=115–148; water loss rate: 1222, 95% CI=1120–1378). Together, these results indicate that the insignificant *P*-values are related to small effects, not limited sample size (that is, a low risk of Type-II error).

**Fig. 2.**
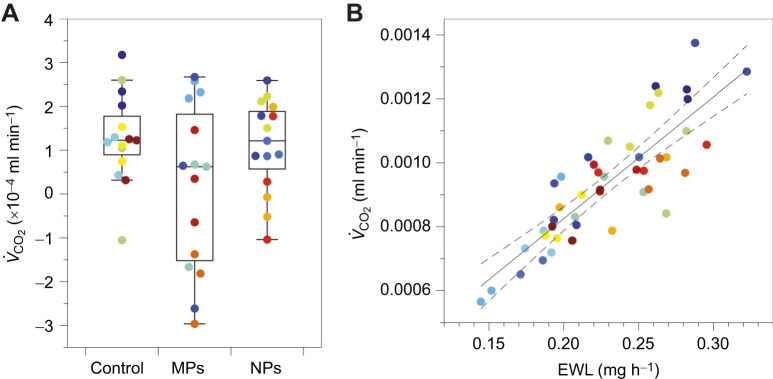
**Metabolic rates were significantly lower and more variable with exposure to MPs.** (A) Exposure to microplastics (MPs) was associated with the lowest metabolic rate (*V̇*_CO_2__) among treatments [control, MPs and nanoplastics (NPs)]. Box plots (median, upper and lower quartiles and 1.5× interquartile range) are shown with point colors representing unique groups of flies. The plots display the adjusted responses, with *V̇*_CO_2__ values corrected for the effects of mass and the main effect of evaporative water loss (EWL) rate (resulting in negative estimates). *Post hoc* tests revealed no differences between pairwise comparisons, though metabolic rate was more variable with MP exposure. (B) There was a positive correlation between EWL rate and volume of carbon dioxide production, accounting for mass and treatment effects. Partial regressions with 95% confidence intervals are shown, with points representing unique groups of flies.

After accounting for water loss rates, we observed a significant effect of treatment with plastic particles (Fig. 2; *P*=0.049; Tables S3 and S4), with MP exposure corresponding to an 11.4% reduction relative to the control and NP exposure corresponding to a 1.8% reduction. We also observed a moderate effect of treatment on metabolic rate (ω^2^=0.13); however, the *post hoc* analyses did not detect significant differences between the control and treatment groups (*P*≥0.197 for all comparisons). The power analysis indicated a sample size of 20 (95% CI=17–22) would be required for 80% power, suggesting that the non-significant results likely reflect limited statistical power. However, the lack of a significant difference between treatments is also likely because MP exposure primarily affected the variability, rather than the mean, of metabolic rate. Specifically, metabolic rates were significantly more variable across treatments (Fig. 2A; Levene test: *P*=0.021), with metabolic rates being 3.2 and 3.5 times more variable than with NP exposure or control, respectively.

**Fig. 3.**
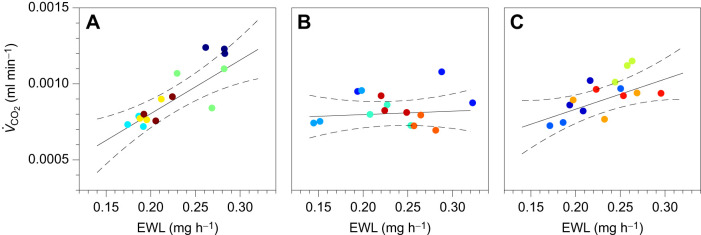
**Exposure to MPs disrupts the relationship between metabolic rate and water loss rate.** (A–C) The relationship between metabolic rate (*V̇*_CO_2__) and EWL rate for each treatment. Experiments revealed a positive relationship between EWL and *V̇*_CO_2__ in the control (A) and NP exposure (C) groups but not in the MP exposure group (B). The adjusted means with 95% confidence intervals are shown with underlying points; colors represent unique groups of flies. The plots display the adjusted responses, with *V̇*_CO_2__ values corrected for the effects of mass.

In agreement with the well-established relationship between respiration and water loss, metabolic rate was significantly and positively associated with water loss rate (Fig. 2B; *P*<0.001). We also observed a large effect of water loss rate on metabolic rate (ω^2^=0.34; Table S5). Moreover, the analysis revealed a significant interaction between water loss rate and treatment on metabolic rate with a moderate effect size (Fig. 3; *P*=0.016, ω^2^=0.19). This interaction indicated that MP exposure significantly disrupted the positive relationship between metabolic rate and water loss, whereas NP exposure did not alter this relationship relative to the control (Fig. 3). *Post hoc* analyses further showed significant differences between the control and MP exposure (*P*=0.026), but not between the control and NP exposure (*P*=0.487) or between NP and MP exposure (*P*=0.267). We found no effects of mass on metabolic rate or water loss rate in any analysis (*P*≥0.67). There are several explanations for how MPs might have disrupted the relationship between metabolic rate and water loss rate.

The results of this study indicate that ecologically relevant exposure to MPs – but not NPs – affected the metabolic rate of *D. melanogaster* when accounting for water loss rate. Based on the significant interaction between treatment and water loss, our experiments revealed that flies with higher water loss rates did not exhibit the expected higher metabolic rates under MP exposure in contrast to control or NP exposure. This suggests that MPs reduce metabolic rates in flies with high water loss rates, while flies with low water loss rates remain unaffected. One possible explanation is that the reduction in metabolic rate is a stress response; organisms are known to lower metabolic rate in response to stressful conditions (Djawdan et al., 1997; Hoffmann and Parsons, 1989). However, in *Drosophila*, the metabolic effects of starvation are often not observed (Hulbert et al., 2004; Harshman and Schmid, 1998). Alternatively, the effect on metabolic rate could be related to physical obstruction by MPs in the gut. Given the relatively large size of MPs, they might interfere with food absorption, leading to metabolic depression. However, if gut obstruction or stress were the primary mechanism, all treated flies would likely experience reduced metabolic rates, not only the flies with higher water loss rates. Further research might quantify the accumulation of microplastics in the gut and assess their impact on food absorption, activity or foraging and reproductive behavior.

Effects on metabolic pathways might explain the disruption of the well-established link between metabolic rate and water loss rate. Both metabolic and water loss rates are highly sensitive to activity, with higher levels typically reflecting increased movement. However, as we found no differences in water loss rate between groups and no clear physiological signs of activity (e.g. irregular spikes), it seems unlikely that overall activity levels differed among the treatments. However, if some chambers of flies were generally more active than others within a treatment, MPs might have affected active metabolic rate more than the resting metabolic rate. This could explain why MP-exposed flies with high water loss rates show reduced metabolic rates, whereas those with lower water loss rates did not. For instance, MP-exposed flies might shift to alternative metabolic substrates (such as shifting from carbohydrates to lipids), resulting in lower carbon dioxide production relative to water loss (Kleiber, 1961; Price and Mager, 2020). Consistent with this pattern, exposure to polystyrene in aquatic organisms appears to disrupt metabolism by initiating the consumption of lipid energy reserves (Jiang et al., 2022). MP dietary exposure has also been linked to overall mitochondrial dysfunction and glucose metabolism disorders, specifically by inhibiting glycolysis and impairing mitochondrial ultrastructures (Hua et al., 2024). Alternatively, dietary polystyrene exposure can disturb the pentose phosphate pathway (Ye et al., 2021), or carbon intermediates may also be shunted into storage compounds (e.g. lipogenesis or polyol synthesis) instead of fully oxidized, reducing CO_2_ output (Arrese and Soulages, 2010). To test these hypotheses, future experiments could examine activity levels, respiratory quotients or respirometry coupled with carbon isotope analyses in flies exposed to MPs and NPs. Additionally, measuring spiracular water loss rates (rather than whole-animal rates) or assessing ventilatory patterns might reveal whether flies are exchanging less air or breathing less frequently (Lehmann et al., 2000; Gibbs et al., 2003). Such studies would help determine whether metabolic dysregulation is a novel consequence of MP exposure.

Understanding how small plastic particles affect organismal physiology requires cross-ecosystem comparisons, particularly between aquatic and terrestrial systems. Research on aquatic organisms revealed that dietary exposure to MPs and NPs can strongly impact metabolism – often resulting in elevated metabolic rates (Yin et al., 2019; Ali et al., 2024) – making metabolism one of the most consistently affected physiological traits (Qiao et al., 2022). However, many of these studies infer metabolic changes from indirect proxies such as gene expression, metabolite profiles, growth or activity levels, rather than measuring metabolic rate directly. Exposure to polystyrene, for example, is commonly associated with altered gene expression and metabolite shifts, reduced growth and lower activity (Qiao et al., 2022; Cong et al., 2019; Yin et al., 2019; Ali et al., 2024). While the mechanisms driving increased metabolism in aquatic species (especially fish) remain uncertain, our results suggest that this pattern does not necessarily extend to terrestrial organisms. Nevertheless, the effects of plastic particles in aquatic organisms appear to be size dependent (Ding et al., 2020), and NPs are not always more toxic than MPs (Qiao et al., 2022), as observed in our study. Together, these comparisons help refine our understanding of how plastic pollution alters organismal metabolism across ecological contexts.

Fruit flies have long served as a valuable model for understanding human exposure, diseases and physiological responses. To date, only a handful of studies have investigated the effects of MPs and NPs on metabolic pathways (Liu et al., 2022; Wang et al., 2021, 2022), and we have a poor understanding of ecologically relevant exposure to small particle plastics on energetics and whole-organism metabolic rate. Consequently, the impacts of MPs and NPs on metabolic rate across a diversity of taxa, including humans, remain poorly characterized. Broadening these investigations to include terrestrial models, such as *Drosophila*, can provide critical insights into how these particles affect digestion and metabolism across diverse species. This research not only deepens our understanding of the potential metabolic challenges posed by MP and NP exposure but also may inform future studies on physiological responses to anthropogenic change, fitness effects on wild insect populations and human health risks.

## Supplementary Material

10.1242/jexbio.250522_sup1 Supplementary information
