# Supplementary material for: Metabolic effects of dietary exposure to polystyrene microplastic and nanoplastic in fruit flies
Source: J Exp Biol. 2025 Oct 13;228(19):jeb250522. doi: 10.1242/jeb.250522 (PMC12579946; doi:10.1242/jeb.250522)
Supplement: Supplementary information [file jexbio-228-250522-s1.pdf]

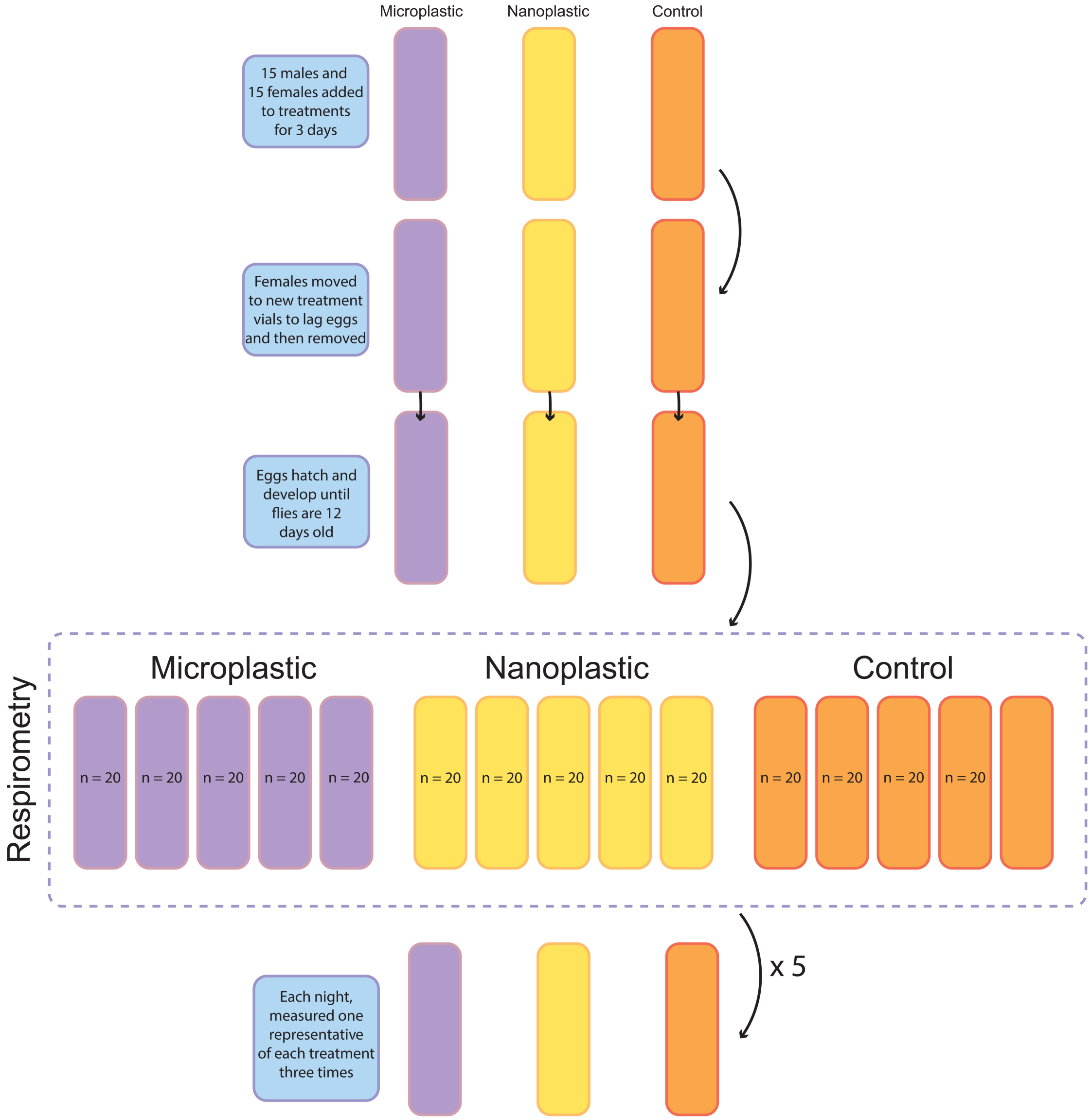

Fig. S1. Flow diagram of experimental protocols. The diagram illustrates how flies were raised under plastic exposure treatments and measured using flow through respirometry. Colors represent treatments (purple = microplastic, yellow = nanoplastic, orange = control). Arrows represent when flies were moved or transferred to the next stage of the experiment. Blue boxes describe the methods in greater detail.

Journal of Experimental Biology • Supplementary information

Table S1. Summary of linear mixed-effects model results for volume of carbon dioxide production. The fixed effects for body mass and treatment (micro- vs. nanoparticle exposure). Reported values include estimated coefficients, standard errors, degrees of freedom, and associated p-values.

Fixed effects	Estimate	Std. Error	*df*	*p*
Intercept	9.419×10^{-4}	2.070×10^{-4}	11	< 0.001
Mass	-2.491×10^{-3}	1.005×10^{-2}	11	0.808
Micro	9.059×10^{-5}	9.059×10^{-5}	11	0.360
Nano	2.526×10^{-6}	9.059×10^{-5}	11	0.978

Table S2. Summary of linear mixed-effects model results for evaporative water loss. The fixed effects for body mass and treatment (micro- vs. nanoparticle exposure). Reported values include estimated coefficients, standard errors, degrees of freedom, and associated p-values.

Fixed effects	Estimate	Std. Error	*df*	*p*
Intercept	0.248	0.055	11	< 0.001
Mass	-1.163	2.683	11	0.672
Micro	0.008	0.024	11	0.749
Nano	0.007	0.025	11	0.791

Journal of Experimental Biology • Supplementary information

Table S3. Summary of linear mixed-effects model results for volume of carbon dioxide production while accounting for evaporative water loss. The fixed effects for body mass, treatment (micro- vs. nanoparticle exposure), and evaporative water loss (EWL). Reported values include estimated coefficients, standard errors, degrees of freedom, and associated p-values.

Fixed effects	Estimate	Std. Error	*df*	*p*
Intercept	0.000128	0.000227	13.7	0.581
Mass	-0.001455	0.006417	10.4	0.825
Micro	0.000654	0.000258	21.0	0.019
Nano	0.000339	0.000297	31.6	0.262
EWL	0.003525	0.000912	16.8	0.001
Micro:EWL	-0.003300	0.001105	21.8	0.007
Nano:EWL	-0.001548	0.001275	32.3	0.233

Table S4. Adjusted marginal means of CO_2 production by treatment, estimated at mean mass (0.020 g) and mean EWL (0.229). Values are estimated marginal means ± SE with 95% confidence intervals (Kenward–Roger df method).

Treatment	Adjusted Mean	SE	*df*	95% CI (Lower, Upper)
Control	0.000907	0.000039	9.22	0.000820 – 0.000994
Micro	0.000804	0.000038	9.19	0.000718 – 0.000891
Nano	0.000891	0.000038	9.20	0.000805 – 0.000978

Table S5. Bootstrap estimates of ω^2 with 95% confidence intervals (1,000 replicates).

**Term**	**Mean ω^2**	**Median ω^2**	**95% CI (Lower)**	**95% CI (Upper)**
Mass	0.000	0.000	0.000	0.209
Treatment	0.127	0.086	0.000	0.248
EWL	0.341	0.196	0.054	0.447
Treatment × EWL	0.187	0.125	0.005	0.300

Journal of Experimental Biology • Supplementary information
